# The elevated serum levels of calcineurin and nuclear factor of activated T-cells 1 in children with Kawasaki disease

**DOI:** 10.1186/s12969-020-0420-8

**Published:** 2020-03-17

**Authors:** Yameng Sun, Jingjing Liu, Zhimin Geng, Yijing Tao, Fenglei Zheng, Ying Wang, Songling Fu, Wei Wang, Chunhong Xie, Yiying Zhang, Fangqi Gong

**Affiliations:** Children’s Hospital, Zhejiang University School of Medicine, No. 3333, Binsheng Road, Hangzhou, 310052 People’s Republic of China

**Keywords:** Kawasaki disease, Coronary artery lesions, Calcineurin, Nuclear factor of activated T-cells

## Abstract

**Background:**

The calcineurin and nuclear factor of activated T-cells (CaN-NFAT) signaling pathway had been found to be associated with Kawasaki disease (KD) susceptibility and coronary artery aneurysm formation as a contributor. To evaluate serum calcineurin (CaN) and nuclear factor of activated T-cells 1(NFAT1) levels in patients with Kawasaki disease (KD).

**Methods:**

Serum levels of CaN and NFAT1 were measured by enzyme-linked immunosorbent assay method in 66 healthy children and 74 KD patients at acute, afebrile and subacute stage.

**Results:**

The serum levels of CaN and NFAT1 increased significantly in the acute stage, and decreased progressively in the afebrile and subacute stage, along with the reduction of C-reactive protein, white blood cells and neutrophil counts. And in the acute stage, the afebrile stage and the subacute stage, the expression of CaN and NFAT1 was upregulated significantly in KD patients compared to that in the healthy control. After the IVIG treatment, the serum levels of CaN and NFAT1 declined significantly in IVIG responders. However, the CaN and NTAT1 levels in the IVIG non-responders declined slowly. And in the afebrile stage, the NFAT1 levels were lower in KD patients with coronary artery lesions (CALs) (268.82 ± 11.96 ng/ml) than those without CALs (285.84 ± 25.13 ng/ml). However, the serum levels of CaN in KD patients with CALs had no significant difference with those in KD patients without CALs.

**Conclusions:**

The specific regulation of CaN and NFAT1 serum levels in the course of KD was suggested that both of them were related in the development of KD.

## Background

Kawasaki disease (KD) is a systemic vasculitis disease that affects small- and medium-sized vessels. The most notably cardiovascular complications caused by KD are coronary artery lesions (CALs). Approximately 20 to 25% of untreated KD patients develop CALs, and KD is now the most frequent cause of childhood acquired heart disease in industrialized countries [1]. Timely treatment with intravenous immunoglobulin (IVIG) can reduce the incidence of coronary artery aneurysms to approximately 4% [2]. Hence, early diagnosis of KD is extremely important and necessary. However, there are no pathognomonic laboratory parameters for diagnosis of KD. Therefore, to make out the etiology and pathogenesis of KD, and specific biomarkers to diagnose KD would be valuable for preventing serious CALs [3].

The calcineurin and nuclear factor of activated T-cells (CaN-NFAT) pathway was first identified in T cells, where NFAT acts as a master regulator of lymphocyte proliferation, differentiation, and development [4, 5]. CaN is a calcium-activated protein phosphatase, which on activation triggers calcium signaling casades including upregulation of inflammatory cytokines like interleukin (IL)-6, IL-10, IL-12, tumor necrosis factor-α (TNF-α) [6, 7]. The elevated serum calcineurin levels has been found in the early onset of coronary artery disease and was associated with the polymorphism at the promoter region on PPP3R1(Protein Phosphatase 3, Regulatory Subunit B, Alpha) [6]. Previous studies have been confirmed that CaN and NFAT plays an important role in lymphocyte development, myocardial hypertrophy, heart failure and coronary artery diseases [6, 8]. NFAT1(NFATp or NFATc2), one of the NFAT transcription factors family, was regulated by Ca^2+^-signaling pathway, with triggering a rapid rise of intracellular Ca^2+^, followed by the activation of phosphatase CaN, NFAT dephosphorylation which can be inhibited by the CaN inhibitors (CNIs) cyclosporin A and FK506, translocation into the nucleus and combination with specific DNA elements in the regulatory regions of target genes [4, 9]. The NFAT1 serum levels has been confirmed to increase significantly in RA patients when compared with control group [10].

Two functional SNPs in CASP3(Caspase 3) and ITPKC (inositol-trisphosphate 3-kinase C) have been identified to be significantly associated with susceptibility to KD [11–13]. ITPKC regulates negatively T-cell and B-cell activation through the Ca^2+^/NFAT signaling pathway, and the C allele may contribute to increased signaling through this pathway, leading to immune hyper-reactivity in KD [12]. The risk allele substitution of CASP3 prevented NFAT from combinating to the DNA sequence surrounding the SNP, resulting in the reduction of CASP3 expression in immune effector cells and thus influencing susceptibility to KD [13]. Moreover, several clinical trials which studied the CaN inhibitors (CNIs, such as cyclosporine A and Tacrolimus) in the treatment of IVIG-resistant KD patients have been found to be effective and well tolerated [14, 15]. However, there are no relevant clinical trials about the serum levels of CaN and NFAT1 with the progression of KD. In order to explore the value of serum CaN and NFAT1 levels in the course of KD, we studied the CaN and NFAT1 sera levels in different stages of KD and healthy control.

## Materials and methods

### Ethical statement

The study was approved by the ethical committee of the Children’s Hospital, Zhejiang University School of Medicine and was based on the institution’s guidelines for human studies. This study conformed to the ethics guidelines of the 1975 Declaration of Helsinki. Written informed consent was obtained from each patient’s parents.

### Patients

In this study, we enrolled 74 fever children (52 boys and 22 girls) diagnosed with KD according to the diagnostic criteria established by the KD Research Committee [16] from November 2015 to March 2017. All patients were treated with IVIG at 1 g/kg/d for 2 days and oral aspirin at 30 to 50 mg/kg/d at the Children’s Hospital, Zhejiang University School of Medicine. After 3 to 5 days of treatment, while the patients’ temperature had returned to normal range, the dosage of aspirin was reduced to 3 to 5 mg/kg/day for 8 to 12 weeks.

There were 9 IVIG-resistant KD patients (7 boys and 2 girls) who were with persistent or recrudescent fever(> 38 °C) after 48 h standard treatment with IVIG, finally accepting another IVIG treatment or glucocorticoids. Nine patients including 7 boys and 2 girls were diagnosed with CALs including transiently dilated coronary arteries and aneurysms (defined as coronary artery z-score > 2) [2] by 2-dimensional Echocardiography. Four patients had CALs in both the left and right coronary arteries, four in the left coronary artery, and one in the right coronary artery. The CALs complications in the seven patients were improved significantly and disappeared in 3 months after standard treatment. Two patients with CALs have persisted more than 9 and 12 months at the last follow-up. In addition, there were 66 healthy children were enrolled into the control group.

### Laboratory analysis

Venous blood samples were respectively collected from the KD patients in three different stages. The acute stage was at the time from the disease diagnosed and admission, and before the IVIG treatment. The afebrile stage was at 3 days after the IVIG treatment and the patients’ temperature had recovered to normal range. The subacute stage was about 14 to 21 days when the patients were first followed up after discharge. The control blood samples were collected when they came for the routine health examination.

Fresh venous blood samples were left to clot at room temperature for 30 min immediately following collection and then centrifuged at 3000 g for 10 min. Then the serum samples were aliquoted and stored at − 80 °C for further analysis. White blood cell (WBC) counts, neutrophil counts (N), platelet counts (PLT), Hemoglobin (Hb), serum C-reactive protein (CRP), erythrocyte sedimentation rate (ESR), N-terminal pronatriuretic peptide (NT-ProBNP), procalcitonin (PCT), alanine aminotransferase (ALT), aspartate aminotransferase (AST), IL-2, IL-4, IL-6, IL-10, TNF-α and Interferon-γ (IFN-γ) were measured by conventional methods in our hospital laboratory. Serum levels of NFAT1 and CaN were quantified by using Enzyme-linked Immunosorbent Assay Kit For NFAT1 (ELISA Kit for Nuclear Factor Of Activated T-Cells, Cytoplasmic 2 (NFATc2); SEL942Hu, cloud-clone corp. Houston, USA) and Calcineurin (CaN) (ELISA Kit for Calcineurin (CaN); SEB323Hu, cloud-clone corp. Houston, USA). The limits of detection for the NFAT1 and CaN ELISA were 5.9 pg/mL and 0.057 ng/mL respectively. The NFAT1 and CaN ELISA kit both show no cross-reactivity or interference with any of the cytokines tested.

### Statistical analysis

All the data were analyzed using SPSS version 22.0 software and presented as mean ± SD except those indicated. Statistical significance between the groups of patients was assessed by using analysis of variance (ANOVA) followed by the least significant difference (LSD) test for multiple comparisons of normal group. Mann-Whitney U test was used for non-parametric data. The differences were considered significant when the *p*-values were < 0.05.

## Results

### Characteristics of the KD patients and healthy children

The demographic and clinical characteristics of these patients are shown in Table 1. In our study, there are 74 KD patients (22 girls and 52 boys) and 66 healthy children (33 boys and 33 girls) enrolled. The median age of KD patients was 26 months (range 3-119 months). There were no significant differences in age and weight between KD patients and healthy control. The duration of fever days was 2–15 days in KD patients. At the acute stage, WBC, N and CRP increased significantly, and then decreased with the course of the disease. Especially after the IVIG treatment, WBC, N and CRP decreased rapidly to the normal. However, at afebrile stage, PLT and ESR was much higher than those in acute stage significantly, and then, decreased gradually to the normal.

**Table 1:** Characteristics of the patients with Kawasaki disease and Healthy control

Characteristics	Kawasaki disease						Healthy Control
	Acute stage	*p* value	Afebrile stage	*p* value	Subacute stage	*p* value	
boys/girls	52/22	0.015					33/33
Age, months (range)	26(3–119)	0.316					33 (2–169)
Weight (kg)	14.7 (6.5–43)	0.416					14.8 (6.8–47)
Days of fever (range)	5.5 (2–15)						–
CaN (ng/ml)	2.97 ± 0.63	<0.001	2.71 ± 0.57**	<0.001	2.46 ± 0.49**^▲▲^	<0.001	1.46 ± 0.33
NFAT1(pg/ml)	305.62 ± 29.29	<0.001	283.77 ± 24.51**	<0.001	259.71 ± 24.42**^▲▲^	<0.001	218.08 ± 32.10
WBC(× 10^9^/L)	14.67 ± 6.13	<0.001	8.5 ± 3.38**	0.355	7.53 ± 2.09**	<0.001	8.9 ± 2.36
NFAT/WBC	27.12 ± 21.58	0.756	38.64 ± 15.55**	<0.001	37.03 ± 11.08**	<0.001	26.27 ± 8.99
CaN/WBC	0.27 ± 0.24	0.004	0.38 ± 0.19**	<0.001	0.35 ± 0.12*	<0.001	0.18 ± 0.07
N(×10^9^/L)	10.12 ± 5.04	<0.001	3.76 ± 2.65**	0.821	2.78 ± 1.34**	<0.001	3.85 ± 1.82
N(%)	66 ± 15	<0.001	41 ± 16**	0.644	36 ± 13**	0.012	42 ± 13
CRP (mg/l)	75.26 ± 52.59	<0.001	13.96 ± 14.83**	<0.001	3.18 ± 2.83**^▲^	0.948	3.15 ± 3.09
PLT(×10^9^/L)	371.23 ± 147.02	0.013	549.30 ± 168.20**	<0.001	361.31 ± 162.99^▲▲^	0.003	320.98 ± 70.44
ESR (mm/h)	71.89 ± 33.75	–	88.81 ± 29.18**	–	18.57 ± 15.27**^▲▲^		–
IL-2(pg/ml)	4.15 ± 1.28	<0.001					3.05 ± 0.89
IL-4(pg/ml)	5.31 ± 5.00	<0.001					2.633 ± 0.69
IL-6(pg/ml)	97.3 ± 139.7	<0.001					11.7 ± 19.87
IL-10(pg/ml)	17.76 ± 21.03	<0.001					4.86 ± 5.7

All *p* values are for patients versus healthy control. *CaN* Calcineurin, *NFAT* nuclear factor of activated T cells, *WBCs* white blood cells, *N* neutrophil, *CRP* C-reactive protein, *PLT* platelet, *ESR* erythrocyte sedimentation rate, *IL-2* Interleukin-2; **p < 0.01 compared with acute stage; ^▲^*p* < 0.05, ^▲▲^*p* < 0.01 compared with afebrile stage

**Fig. 1:**
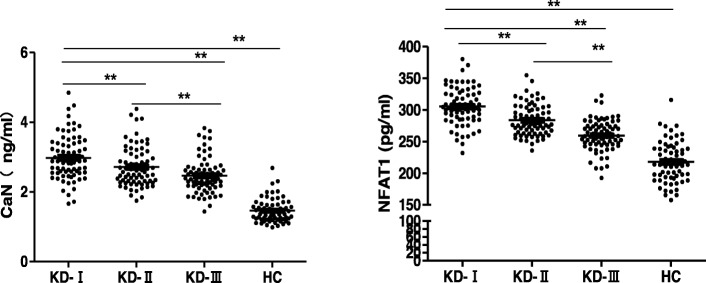
The serum levels of CaN and NFAT1 in patients with KD and healthy control. KD-I: The acute stage; KD-II: The afebrile stage; KD-III: The subacute stage; HC: The healthy control. ** *p* < 0.01

The serum levels of NFAT1 and CaN increased significantly at the acute stage of KD when compared with control group. At the afebrile stage and subacute stage, both NFAT1 and CaN decreased gradually with the process of KD, but remained higher than that of the healthy control (Fig. 1).

### Serum levels of CaN and NFAT1 in IVIG responding and non-responding KD patients

The serum levels of CaN and NFAT1 in IVIG responding and non-responding KD patients are shown in Table 2. In our study, there were 9 KD patients (7 boys and 2 girls) who were not sensitive to the IVIG treatment. After the IVIG treatment, the serum levels of CaN and NFAT1 declined significantly in IVIG responders. However, the CaN levels in the IVIG non-responders declined slowly, in which there were no significant differences between the acute, afebrile and subacute stages in IVIG non-responders KD patients. The same results can also be found in the NFAT levels of IVIG non-responders KD patients when comparing the NFAT levels between the acute afebrile and subacute stages. Nevertheless, if comparing the CaN and NFAT1 levels between IVIG responder and non-responders KD patients, none of them showed significant differences in the acute, afebrile and subacute stages.

**Table 2:** Serum levels of CaN and NFAT1 in IVIG responding and non-responding patients with KD

Characteristics	stage	IVIG responders (*n* = 65)	IVIG non-responders(*n* = 9)	*p* value
Boys/girls		45/20	7/2	0.716
CaN (ng/ml)	Acute stage	3.02 ± 0.64	2.67 ± 0.50	0.125
	Afebrile stage	2.74 ± 0.57^▲^	2.48 ± 0.48	0.163
	Subacute stage	2.47 ± 0.49^▲^*	2.34 ± 0.54	0.354
NFAT1(pg/ml)	Acute stage	304.35 ± 29.35	314.84 ± 28.76	0.317
	Afebrile stage	283.26 ± 24.81^▲▲^	287.94 ± 22.98	0.726
	Subacute stage	258.43 ± 24.32^▲▲^**	270.27 ± 24.09^▲▲^	0.145

^▲^*p* < 0.05, ^▲▲^*p* < 0.01 compared with acute stage. *p < 0.05, **p < 0.01 compared with afebrile stage

### Serum levels of CaN and NFAT1 in KD patients with or without CALs

The serum levels of CaN and NFAT1 in KD patients with or without CALs are shown in Table 3. The results showed that the serum levels of CaN and NFAT1 descended significantly after treated with IVIG in KD patients with or without CALs. The serum levels of CaN had no significant difference between those KD patients with or without CALs. The serum levels of NFAT1 in KD patients with CALs were lower than those in KD patients without CALs at all of three stages. Especially at afebrile stage, the NFAT1 levels of KD patients with CALs were significantly lower than those of KD patients without CALs (268.82 ± 11.96 pg/ml vs 285.84 ± 25.13 pg/ml, *P* = 0.003). In addition, we found that IL-4, IL-6 and IL-10 levels in KD patients with CALs were much lower than that in KD patients without CALs.

**Table 3:** Serum levels of CaN and NFAT1 in Kawasaki disease with or without coronary artery lesions

Characteristics	stage	KD with CALs(*n* = 9)	KD without CALs(*n* = 65)	*p* value
Boys/girls		7/2	45/20	0.716
CaN (ng/ml)	Acute stage	3.13 ± 0.52	2.95 ± 0.65	0.444
	Afebrile stage	2.77 ± 0.46	2.70 ± 0.58^▲^	0.743
	Subacute stage	2.50 ± 0.44^▲^	2.45 ± 0.50*^▲▲^	0.226
NFAT1(pg/ml)	Acute stage	297.46 ± 13.84	306.75 ± 30.73	0.135
	Afebrile stage	268.82 ± 11.96^▲▲^	285.84 ± 25.13^▲▲^	0.003
	Subacute stage	250.43 ± 20.15*^▲▲^	261.00 ± 24.81**^▲▲^	0.226
IL-2(pg/ml)	Acute stage	3.83 ± 1.28	4.20 ± 1.28	0.442
IL-4(pg/ml)	Acute stage	3.5 ± 1.15	5.61 ± 5.33	0.013
IL-6(pg/ml)	Acute stage	35.19 ± 29.14	107.8 ± 148.28	0.002
IL-10(pg/ml)	Acute stage	8.30 ± 6.99	19.36 ± 22.21	0.006

^▲^*P* < 0.05, ^▲▲^*P* < 0.01 compared with patients at acute stage

*P < 0.05, **P < 0.01 compared with patients at afebrile stage

### Serum levels of CaN per WBC at different stages of KD patients

**Fig. 2:**
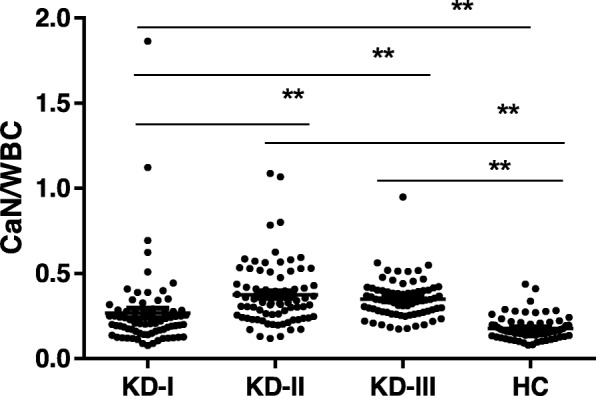
CaN level per WBC at different stage of KD treated with intravenous immunoglobulin. KD-I, acute stage; KD-II, afebrile stage; KD-III, subacute stage; HC, healthy control. **p < 0.001

In order to figure out whether the CaN serum level was due to the prominent increasing WBC. We compared the CaN level by standardizing to WBC (Table 1 and Fig. 2). The results showed that the CaN level per WBC was lower in acute stage of KD than that in afebrile and subacute stage. When compared with healthy control, the CaN level per WBC in acute stage was much higher. In the afebrile and subacute stage, along with the recovery of inflammation, the WBC decreased rapidly, but the CaN level didn’t decrease synchronously. Therefore, the CaN serum level per WBC was much higher than that in the acute stage of KD and the healthy control.

## Discussion

According to our study, in the acute stage of KD, the serum levels of CaN and NFAT1 increased to peak with the course of disease. After the treatment of IVIG, the CaN and NFAT1 levels decreased gradually at the afebrile stage and subacute stage, along with the reduction of CRP, WBC and neutrophils. The serum levels of CaN and NFAT1 at the subacute stage were still significantly higher than those in the healthy control, which means that the CaN and NFAT1 levels didn't return to normal at the subacute stage. Our data indicated that CaN and NFAT1 signal pathway could play a potential role in the pathophysiology of KD. In addition, it suggests that the reduction of inflammation is associated with the reduction of NFAT1 sera levels. By using the CNIs to block the NFAT translocation to the nucleus, the inflammation of KD at acute stage could be relieved.

The CaN-NFAT signal pathway as a contributor had been found to be associated with KD susceptibility and CALs formation, growing out of 3 functional single nucleotide polymorphisms (SNPs) in ITPKC, CASP3 and SLC8A1(solute carrier family 8, member 1, a sodium/calcium exchanger encoding NCX1) gene [11, 12, 17]. Previous studies had demonstrated that T effector memory cells with a proinflammatory phenotype and macrophages were involved in the acute stage of KD, and pathology findings of coronary artery autopsy tissues showed that the coronary arterial wall was infiltrated by CD8+ T cells [18, 19]. The flow cytometry results in the IVIG-responsiveness KD showed that extreme CD8 + T cell activation and an disequilibrium in CD8 + T cell activation and inhibition were important in the pathogenesis of KD [20]. The CaN-NFAT signal pathway had been confirmed to participate in the T-helper-cell differentiation, T-cell activation and T-cell tolerance, but also participate in the transcriptional program of CD8+ T cell exhaustion [5, 9, 21]. NFAT1 can cooperate with AP-1 to induce the expression of inflammatory cytokines [9, 21]. Moreover, NFAT1 controls the exhaustion of CD8+ T cell by inducing the inhibitory surface receptors when not cooperate with AP-1 [21]. These researches may suggest that KD susceptibility, CALs formation and response to IVIG are associated with CaN-NFAT signaling pathway.

CaN is a key phosphatase in immunity, many of which are facilitated by dephosphorylation of NFAT and translocation of NFAT from the cytoplasm to the nucleus to regulate cell proliferation, differentiation and development [22]. In our study, the serum levels of CaN were significantly increased in the acute stage of KD, coincident with the NFAT1 levels, which suggests that the pathway is activated. Once the CaN-NFAT pathway is activated, the NFAT is dephosphorylated and translocated to the nucleus, which modify immune cell maturation, cytokine production and release such as IL-2, IL-4, IL-6 and cell survival [5]. In our study, we also found the serum of IL-2, IL-4, IL-6, IL-10 were increasing at acute stage of KD when compared with healthy control. Previous studies have reported that in the early stage of KD, the TNF, IL-1 and IL-6 signaling pathways were activated and the activated circulating neutrophils increased significantly, which is suggested the abnormal activation of innate immunity [23]. Moreover, recent studies have shown that CaN-NFAT signaling also plays a vital role in innate cell activation, including the activation of dendritic cells (DCs) and natural killer cells (NK cells) which is primed by DCs and enhanced by DCs production of IL-2 [5]. Besides, CaN-NFAT signaling regulated the expression of IL-6, IL-10, IL-12 and TNF-α in macrophages, most of which have been confirmed to be involved in the pathogenesis of KD [24–27]. On the other hand, in the first week of KD onset, proinflammatory and regulatory T cells are found in the circulation, which suggests the activation of adaptive immunity [19]. After the treatment of IVIG, the increase of the regulatory T-cell population in the peripheral blood is related with defervescence and clinical improvement [28]. In our data, the levels of CaN and NFAT1 decreased significantly at the afebrile and subacute stage after the IVIG treatment, suggesting that the CaN-NFAT signaling pathway plays a potential role in onset of KD, and along with the reduction of CaN and NFAT1 sera levels, the fever is resolved and inflammation is alleviated.

Although the serum levels of CaN in KD patients with CALs had no significant difference from those in KD patients without CALs due to limited sample size, the serum levels of NFAT1 in KD patients with CALs were lower significantly than those in KD patients without CALs at afebrile stage, which suggest that NFAT1 levels decline much rapidly after the standard IVIG treatment in the KD patients with CALs than those without CALs. A recent study has confirmed that homozygous KD patients for the SLC8A1 A (risk) allele of rs13017968 were more likely to develop coronary artery aneurysms [17], which suggested the activation and importance of calcium signaling pathway in KD patients with CALs. These findings seemed to be consistent with our study, suggesting the availability of using CNIs in KD with CALs. Blocking the calcium signaling pathway by CNIs, resulting in blocking the dephosphorylation of the transcription factor NFAT may reduce acute inflammation and CALs in KD patients.

According to our data, after the IVIG treatment, the serum levels of CaN and NFAT1 declined significantly in IVIG responders. However, the CaN and NTAT1 levels in the IVIG non-responders declined slowly, suggesting that the CaN and NFAT1 levels in IVIG resisitant KD patients cannot be decreased effectively by using IVIG. Furthermore, we found that at the acute and subacute stage, the serum levels of NFAT1 were little higher in the IVIG non-responders than those in IVIG responders (although there were no significant differences with the limited sample size), which suggest that the immune response in IVIG non-responders may be more severe than in IVIG-responders. Clinical trials have confirmed that CaN inhibitors are effective and have no serious adverse effects in the treatment of IVIG-resistant KD patients [14, 15]. A recent study which is a randomised controlled, open-label, blinded-endpoints, phase 3 trial aiming to compare the efficacy of IVIG plus CNIs or IVIG alone when treating the KD patients with high risks for IVIG resistance, reported that combination therapy with IVIG and CsA can decrease the incidence of CALs [29]. Therefore, the mechanisms of CaN inhibitors in treating KD patients especially in IVIG resistance patients may be because of rapid defervescence and resolution of inflammation by specifically blocking the abnormal activation of CaN-NFAT pathway, sequentially suppressing the undesired innate immune and adaptive immune responses, including inhibiting the production of proinflammatory cytokines and a reduction of circulating activated CD4+ and CD8+ T effector memory cells.

Previous studies have reported the WBC and activated circulating neutrophils increased significantly and also the apoptosis of circulating neutrophils in acute stage of KD was delayed [30]. Therefore, there is a hypothesis which needs to be confirmed-whether the elevated CaN and NFAT1 serum levels was caused by high WBC. As is known to all, NFAT2 and NFAT4 not NFAT1 were present in neutrophil, which is suggested that the increased NFAT1 level was not caused by neutrophil. In addition, previous study has reported that there were no significant increase in mononuclear cells between KD and control groups or in acute and convalescent stage of KD [23], which is suggesting the reason causing the increasing WBC in KD was the increasing Neutrophils. However, the subgroup of lymphocytes and monocytes/macrophages were differentially regulated in acute stage of KD. Taken together, it is indicated that not the increased amount of WBC but the differential regulation of lymphocytes and monocytes/macrophages subgroups caused the increase of NFAT1 serum levels.

CaN as a heterodimeric serine/threonine phosphatase enzyme can be found in all tissues except γ isoform [22]. Thus, we compared the CaN level by standardizing to WBC. According to our results, the CaN level increased in the acute stage and gradually declined after treatment by IVIG. The WBC rised rapidly at acute stage with the delayed neutrophils apoptosis and declined rapidly with the promotion of neutrophils apoptosis due to the IVIG treatment. The CaN level per WBC were much higher than that in the acute stage of KD and the healthy control. The relative non-synchronization with WBC as they evolve from acute to chronic stage suggested that the elevated CaN serum level was not caused by high WBC. However, previous studies have reported that NFAT was involved in the regulation of apoptosis [31]. According to our results, after IVIG treatment, the NFAT1 serum levels shifted from high to close to normal. At the same time, after IVIG treatment, the apoptosis of neutrophils shifted from delayed to be promoted, appearing to be the same trend with the NFAT1 levels. Cytokines such as IL-1β, IL-6, IFN-γ and TNF-a which can be regulated by CaN/NFAT signaling pathway can inhibit neutrophil apoptosis [30]. Whether the elvated NFAT1 in acute stage of KD resulted in delayed neutrophils apoptosis need to be further confirmed.

## Conclusions

In conclusion, CaN and NFAT1 are both increased at the acute stage of KD, and declined gradually at the afebrile stage and subacute stage after the treatment of IVIG, along with the resolution of inflammatory and clinical improvement. The specific regulation of CaN and NFAT1 serum levels in the course of KD was suggested that both of them were related in the development of KD.

## Data Availability

Data can be requested from the corresponding author.

## Abbreviations

ANOVA: Analysis of variance
CaN: Calcineurin
CASP3: Caspase-3
CNIs: CaN inhibitors
CRP: C-reactive protein
DCs: Dendritic cells
ELISA: Enzyme-linked immunosorbent assay
ESR: Erythrocyte sedimentation rate
Hb: Hemoglobin
IFN-γ: Interferon-γ
IL-6: Interleukin-6
ITPKC: Inositol 1,4,5-trisphosphate 3-kinase C
IVIG: Intravenous immunoglobulin
KD: Kawasaki disease
LSD: Least significant difference
N: Neutrophil counts
NCX1: Na^+^ −Ca^2+^ exchanger isoform 1
NFAT1: Nuclear factor of activated T-cells 1
NK cells: Natural killer cells
PLT: Platelet counts
SLC8A1: Solute carrier family 8, member 1
SNPs: Single nucleotide polymorphisms
TNF-α: Tumor necrosis factor-α
WBC: White blood cell
